# One Polish Center’s Experience of Catheter Ablation for Cardiac Arrhythmias in Dogs

**DOI:** 10.3390/vetsci13080760

**Published:** 2026-07-30

**Authors:** Agnieszka Noszczyk-Nowak, Piotr Frydrychowski, Justyn Gach, Zuzanna Wojtczak, Alicja Cepiel-Kośmieja, Artur Fuglewicz, Krzysztof Nowak

**Affiliations:** 1 Department of Internal Medicine and Clinic of Diseases of Horses, Dogs and Cats, Faculty of Veterinary Medicine, University of Environmental and Life Sciences, 50-366 Wroclaw, Poland; piotr.frydrychowski@upwr.edu.pl (P.F.); justyn.gach@upwr.edu.pl (J.G.); zuzanna.wojtczak@upwr.edu.pl (Z.W.); alicja.cepiel@upwr.edu.pl (A.C.-K.); 2 Cardiac Lab, Jozef Strus City Hospital, 61-285 Poznan, Poland; fuglewiczartur@gmail.com; 3 Institute of Heart Diseases, Medical University of Wroclaw, 50-556 Wroclaw, Poland

**Keywords:** arrhythmias, dogs, electrocardiography, radiofrequency catheter ablation

## Abstract

Radiofrequency catheter ablation is a minimally invasive electrophysiological procedure that utilizes radiofrequency energy to induce targeted thermal injury within arrhythmogenic myocardial tissue, thereby eliminating the substrate responsible for rapid and irregular cardiac rhythm disturbances. Radiofrequency ablation is a method widely used in human medicine for the permanent treatment of arrhythmias. In veterinary medicine, there are only a few centers worldwide that use this treatment method in dogs. This article presents the experiences of a Polish center in treating supraventricular and ventricular arrhythmias using this method. The first electrophysiological study and ablation were performed using an analog electrophysiological system; subsequent procedures were performed with the aid of a computerized system, and the last three procedures were performed using a 3D electroanatomical system. A total of 14 radiofrequency ablation procedures were performed on dogs weighing 10 to 33 kg, aged 9 months to 9 years, of various sexes and breeds. Fifty percent of the dogs that underwent ablation had both clinical and echocardiographic signs of tachycardia-induced cardiomyopathy. Three dogs required a re-ablation procedure. Complete elimination of clinically significant arrhythmias was achieved in 13 of 14 dogs, while one dog showed partial procedural success. No significant or permanent complications were observed in any of the dogs. Radiofrequency ablation procedures for arrhythmias are effective and safe.

## Simple Summary

Cardiac arrhythmias in dogs can cause clinical signs and, when rapid and persistent, may lead to reversible heart muscle dysfunction. Radiofrequency catheter ablation is a minimally invasive procedure in which heat energy is delivered through a catheter to the small area of heart tissue responsible for the abnormal rhythm. Although this treatment is well-established in human medicine, it remains available in only a few veterinary centers. This study describes the experience of a Polish center with radiofrequency catheter ablation for both upper- and lower-chamber arrhythmias in dogs. Fourteen procedures were performed on dogs of different breeds, sexes, ages, and body weights. Half of the dogs showed pre-operative clinical and heart ultrasound evidence of rhythm-related heart muscle dysfunction. Three dogs required a second procedure. No major or permanent complications were observed. Over time, the procedures were performed with increasingly advanced mapping systems, including three-dimensional electroanatomical mapping.

## 1. Introduction

Radiofrequency catheter ablation (RFCA) is a minimally invasive therapeutic procedure used to treat cardiac arrhythmias, preceded by an electrophysiological study (EPS), which involves recording intracardiac signals and stimulating specific areas of the heart. This ablation procedure involves destroying the heart tissue that is the source of the arrhythmia through controlled heating using high-frequency current [1,2]. RFCA is highly effective in treating both benign and life-threatening atrial and ventricular arrhythmias. It is a permanent method with the longest history of use compared to other ablation techniques in both human medicine and clinical veterinary medicine. RFCA is the primary method of ablation in veterinary medicine for dogs [3,4,5,6,7,8,9,10,11,12,13,14,15,16,17,18,19,20,21,22,23,24], especially in animals where pharmacological treatment is ineffective or impossible. This method is used for both relatively simple ablations, such as accessory pathway ablation [3,4,5,6,7,8], and complex ventricular arrhythmias [11,20,21]. The RFCA procedure is associated with a relatively low risk of complications; however, it is not entirely devoid of procedural risk [3,4,5,6,7,8,9,10,11,12,13,14,15,16,17,18,19,20,21,22,23,24].

The first RFCA procedures were performed using analog recording of intracardiac signals, and electrodes were inserted into the heart chambers under fluoroscopic imaging. Subsequently, computerized systems for recording and processing intracardiac signals were introduced, which significantly improved the quality of the recordings. The next stage in the development of EPS techniques involves systems for three-dimensional (3D) imaging of the heart. Three-dimensional electroanatomical mapping (3D EAM) is a modern imaging technique used during cardiac ablation procedures, including those performed in dogs [20,21,22,23,25,26], which significantly increases the precision, safety, and effectiveness of the procedure. The use of 3D EAM enables the creation of an accurate, personalized model of the patient’s heart. 3D EAM allows for the creation of 3D potential, impedance, propagation, or temporal maps in real time with simultaneous recording of intracardiac signals. The visualization of the catheter and its orientation in space is based on a magnetic field or impedance, or both methods simultaneously.

Although RFCA is an established treatment for selected arrhythmias in human electrophysiology, its use in veterinary medicine is still limited to a small number of highly specialized centers. In dogs, the most substantial clinical experience has been reported for supraventricular tachyarrhythmias, particularly those mediated by atrioventricular accessory pathways (APs). In the largest published cohort, Wright et al. described 89 dogs referred for RFCA of AP-mediated tachycardia. Most pathways were right-sided, accounting for 91.7% of APs, with the right posteroparaseptal region being the most common location, followed by the right free wall. Tachycardia-induced cardiomyopathy was identified in 46.1% of dogs and improved or resolved in most affected patients after ablation. Long-term elimination of AP conduction was achieved in 98.8% of dogs in which RFCA was performed. Recurrence was documented in three dogs, all of which were treated successfully with a second procedure, resulting in a reported single-procedure long-term success rate of 95% and an overall long-term success rate of 98.8% after repeat ablation when needed [17].

Comparable outcomes were reported in the Italian experience described by Santilli et al., which included 76 consecutive dogs and 83 accessory pathways treated between 2008 and 2016. Most dogs had a single AP, although multiple pathways were identified in 7.9% of cases. As in the study by Wright et al., the vast majority of pathways were right-sided, representing 96.4% of APs, whereas left-sided pathways were uncommon and accounted for only 3.6%. The predominant arrhythmia mechanism was orthodromic atrioventricular reciprocating tachycardia (OAVRT), diagnosed in 92.1% of dogs, while permanent junctional reciprocating tachycardia was reported in 6.5%. RFCA was attempted in 97.4% of dogs and was acutely successful in all treated cases. During follow-up, recurrence was observed in 7.7% of dogs within 18 months; these patients underwent a second procedure, which was ultimately successful. Major complications were uncommon but clinically relevant, with pacemaker implantation required in 2.6% of dogs [18].

Catheter ablation has also been explored in other canine supraventricular arrhythmias, although the available evidence is based on smaller cohorts. Santilli et al. evaluated 16 dogs with symptomatic focal atrial tachycardia and identified 19 focal atrial tachycardias. Most ectopic foci originated from the right atrium, including the crista terminalis, triangle of Koch, tricuspid valve annulus, interatrial septum, and right auricle, while 37% were localized to the pulmonary veins. This study helped define the electrophysiological characteristics and anatomical distribution of focal atrial tachycardia in dogs and underscored the importance of detailed endocardial mapping for accurate localization [9].

Reports of RFCA for atrial flutter in dogs are also limited but informative. Typical cavotricuspid isthmus-dependent atrial flutter was treated successfully in two dogs, in which bidirectional cavotricuspid isthmus block was achieved, and follow-up Holter monitoring confirmed resolution of the arrhythmia [10]. In a separate series of five dogs with atypical atrial flutter, electrophysiological mapping identified right atrial macro-reentrant circuits involving either a right septal isthmus or a right atrial free-wall isthmus. Linear RFCA interrupted the macro-reentrant circuit acutely in all dogs. Long-term rhythm control, however, was more variable: three dogs remained free of recurrence at 18 months, one experienced recurrence within 15 days, and one developed persistent atrial fibrillation two months after the procedure [15]. Cranial vena cava flutter has also been described in four dogs, in which linear ablation extending from the right atrial wall to the distal cranial vena cava produced permanent interruption of isthmic conduction at six-month follow-up [19].

In contrast to the relatively strong evidence available for supraventricular tachycardias, experience with RFCA for ventricular arrhythmias in dogs remains sparse. Santilli et al. reported five English Bulldogs with ventricular tachycardia associated with localized right ventricular outflow tract enlargement. In one of these dogs, electrophysiological mapping identified two sites of origin within the right ventricular outflow tract, located in the caudal free-wall and cranial septal regions, and RFCA of the ventricular tachycardia substrate was performed [11]. More recently, Crooks et al. described electroanatomic mapping-guided RFCA in five Boxer dogs with symptomatic or medically refractory ventricular tachycardia. Sustained ventricular tachycardia was inducible in three dogs, low-voltage areas consistent with electroanatomic scar were found in two, and the suspected arrhythmogenic substrate involved the right ventricular outflow tract or cranial right ventricle. Following RFCA, collapse was no longer reported in any dog, and the number of ventricular tachycardia runs decreased in all cases; three dogs had no VT runs after the procedure. The effect on overall ventricular premature complex burden was less consistent, increasing in three dogs and decreasing in two, with near-complete arrhythmia resolution achieved in only one dog. These findings support the technical feasibility of ventricular arrhythmia mapping and ablation in dogs, while also illustrating the complexity and variability of ventricular arrhythmia substrates in this species [21].

Another report described a dog with a drug-refractory ventricular arrhythmia, mainly presenting as ventricular bigeminy, in which no structural heart disease was identified. Three-dimensional electroanatomical mapping localized the endocardial focus to the basal-anterior portion of the left ventricular septum, close to the conduction system. Two RF applications were delivered at this site. The dog showed marked clinical improvement, and follow-up 48 h Holter monitoring demonstrated complete resolution of the arrhythmia [20].

The technical feasibility of such procedures was further addressed by Hellemans and colleagues. Their study showed that 3D electroanatomical mapping can be performed in dogs and may help reduce the use of fluoroscopy. However, the authors also emphasized that procedures involving left-sided cardiac access and transseptal puncture are associated with a real risk of complications. This is particularly important in the context of ventricular arrhythmia ablation, where accurate mapping is essential to identify the site of origin of VT or VPCs, especially when the focus is located in the left ventricle, the RVOT, the interventricular septum, or near the conduction system [25].

Additional experience from a United Kingdom referral center further reflects the gradual expansion of canine electrophysiology and RFCA beyond the early Italian and North American reports. Wringe et al. retrospectively reviewed 44 dogs undergoing electrophysiological study and RFCA for supraventricular tachycardia at Davies Veterinary Specialists between 2014 and 2024. Suspected OAVRT was the most common pre-procedural rhythm diagnosis, identified in 28 dogs, while smaller numbers of patients were diagnosed with focal atrial tachycardia, atrial flutter, atrial fibrillation, wide-complex tachycardia, or other rhythm disturbances. Although the primary aim of that study was to describe anesthetic management and peri-operative complications rather than long-term arrhythmia outcomes, it provides further evidence that EP/RFCA procedures are now being performed in specialized veterinary centers in different countries [24].

Overall, the current literature suggests that RFCA is best established in dogs with AP-mediated tachycardias, where published series report high acute and long-term success rates and a relatively low need for repeat ablation. For focal atrial tachycardia, atrial flutter, and especially ventricular arrhythmias, the evidence remains more limited and is largely derived from small case series or individual reports. The present study therefore adds to the still-developing veterinary literature by describing the experience of a single Polish center performing electrophysiological studies and RFCA in dogs with both supraventricular and ventricular arrhythmias. In our study, we focused on the results of clinical, electrocardiographic, echocardiographic, and electrophysiological examinations in a series of consecutive cases of dogs with tachyarrhythmias who underwent RFCA and presented the long-term treatment outcomes in a single-center clinical population of dogs.

## 2. Materials and Methods

A retrospective analysis was conducted of the medical and veterinary records of 14 dogs that underwent RFCA at the Department of Internal Medicine and Clinic of Diseases of Horses, Dogs and Cats between 2010 and 2026. All dogs underwent a comprehensive physical examination, a 5 min 12-lead electrocardiogram, a 24 h Holter ECG, chest radiography, echocardiography, and electrophysiological mapping. Clinical signs associated with the presence of tachycardia as well as antiarrhythmic therapy and treatment for heart failure were included in the descriptive statistics because the sample size was too small for statistical analysis.

Before performing EPS, antiarrhythmic drugs were discontinued for at least 5 half-lives [18]. The EPS study and RFCA were performed under general anesthesia according to the protocols described by Santilli [18]. The animals were positioned in dorsal recumbency; venous and arterial access was established using the true Seldinger technique; and then, under fluoroscopic and intracardiac ECG guidance, multiple diagnostic (1 to 3) and ablation catheters were inserted into the coronary sinus in all cases and additionally positioned in the right atrium, right ventricle, or left ventricle according to the type of arrhythmia being treated. In the case of the 3D EAM CARTO system used in three dogs, the diagnostic and ablation electrodes were placed without the use of fluoroscopy. Programmed electrical stimulation was performed in the high right atrium and right ventricular apex. In selected cases, left ventricular mapping was also performed. The final diagnosis of arrhythmia was based on an analysis of intracardiac electrograms, as well as 12-lead surface ECGs and intracardiac recordings.

After the procedure, dogs were observed for 6–8 h. During this period, heart rate, oxygen saturation, ECG, non-invasive blood pressure, body temperature, and respiratory rate were assessed. A 12-lead electrocardiogram was performed at 7 days and at 1, 6, and 12 months after the ablation, and Holter monitoring was recommended at 1, 6, and 12 months after the procedure. Holter recordings were analyzed to assess arrhythmia recurrence.

Specific arrhythmias were diagnosed using the following criteria. Focal atrial tachycardia (FAT) was defined as a regular narrow-QRS tachycardia of supraventricular origin with an atrial rate of 210–330 bpm, a long RP′ interval, and an RP′/P′R ratio ≥0.7. P′-wave morphology varied with focus location; it was most commonly positive in the inferior leads and could overlap the T wave, producing the characteristic “camel sign.” OAVRT was defined as a paroxysmal, regular narrow-QRS tachycardia with an atrial rate of 190–300 bpm, a short RP′ interval, an RP′/P′R ratio ≤0.7, and retrograde P′ waves located within the ST segment and negative in the inferior leads. Ventricular ectopy was identified by QRS complexes >70 ms, wide T waves of opposite polarity to the main QRS deflection, and morphology differing from sinus beats. Ventricular complexes were classified according to timing as premature, interpolated, or escape complexes and according to pattern as isolated complexes, bigeminy, or trigeminy. Ventricular tachycardia was defined as a wide-QRS tachycardia with a morphology different from sinus rhythm, a ventricular rate >180 bpm, and at least four consecutive ventricular complexes, according to Santilli et al. [27].

Tachycardia-induced cardiomyopathy (TICM) was diagnosed on the basis of echocardiographic examination. Assessment of the left ventricle included measurements of left ventricular dimensions in diastole and systole, as well as fractional shortening derived from M-mode. Left ventricular end-diastolic volume, end-systolic volume, and ejection fraction were calculated using Simpson’s method of disks. Evaluation of the right ventricle was performed both subjectively and objectively, based on parameters of systolic function (tricuspid annular plane systolic excursion [TAPSE] and tissue Doppler imaging-derived systolic velocity [TDI S’]) and diastolic function, including right ventricular end-diastolic area (RVEDA). The size of the right and left atria was assessed on the basis of measurements along the long and short axes (LA/Ao ratio).

Follow-up echocardiographic examinations were performed at 1, 6, and 12 months after the catheter ablation procedure. Echocardiographic improvement was defined as normalization of right and left ventricular volumes, normalization of right and left atrial size, and normalization of normal systolic function of both ventricles. In patients with right-sided heart failure, clinical improvement was additionally assessed by the absence of recurrent ascites.

Antiarrhythmic therapy was considered ineffective when stabilization of the patient’s condition was not achieved despite monotherapy or combination therapy together with treatment for congestive heart failure (CHF), as reflected by persistent or recurrent CHF. Treatment was also considered ineffective when antiarrhythmic monotherapy or combination therapy failed to provide a satisfactory reduction in arrhythmia burden, defined as a reduction of at least 50%, and when the arrhythmia adversely affected the dog’s quality of life, including apathy, exercise intolerance, or reluctance to exercise. Twenty-four-hour Holter monitoring was performed to assess treatment efficacy within 2–4 weeks after initiation or modification of therapy, depending on the type of arrhythmia, treatment protocol, and the owners’ ability to attend follow-up examinations.

During the differential diagnostic work-up of cardiac arrhythmias and echocardiographic abnormalities, the dogs underwent laboratory testing, including complete blood count and serum biochemistry analyses, as well as measurement of cardiac troponin I concentrations to exclude active myocarditis. If the medical history and clinical signs were suggestive of an infectious disease, appropriate diagnostic investigations were performed to rule out an infectious etiology.

Inclusion criteria for the ablation procedure included the presence of cardiac arrhythmias amenable to catheter ablation, with a minimum body weight allowing for venous and arterial vascular access (typically ≥5 kg), canine species; lack of reduction in arrhythmia frequency or severity despite antiarrhythmic therapy administered as monotherapy or combination therapy; presence of hemodynamic consequences (e.g., echocardiographic features consistent with TICM); and the presence of clinical symptoms resulting from arrhythmias (e.g., syncope, RCHF, exercise intolerance).

Exclusion criteria consisted of: advanced underlying congenital or acquired heart disease considered to be the primary cause of the arrhythmia; active myocarditis; the presence of severe, uncontrolled systemic disease and/or neoplastic disease associated with an expected short survival time; and species other than dogs were excluded.

## 3. Results

### Population Study

A total of 14 dogs were included in the study (nine males and five females) with an M/F ratio of 1.8, a median age of 2.5 years (range 9 months to 9 years), and a mean weight of 27.7 ± 8.5 kg. The most represented breed was Labrador Retriever (4/14, 28.5%) (Table 1). None of the patients referred for EPS and catheter ablation showed evidence of active infection or active myocarditis, as assessed on the basis of cardiac troponin I concentration.

Ten out of 14 (71.4%) dogs were presented with incessant supraventricular tachycardia. Seven out of 14 (50%) dogs demonstrated echocardiographic evidence of TICM. Four dogs presented with signs of right-sided congestive heart failure (RCHF), including ascites, and three were in stage B2 TICM (without RCHF). Only one dog had a congenital heart defect—mild tricuspid valve dysplasia. Echocardiographic abnormalities resolved between 3 and 6 months after the procedure. Patients with systolic dysfunction received pimobendan prior to ablation, which was discontinued after normalization of systolic function. Patients with right ventricular failure were treated with loop diuretics—furosemide or torasemide; treatment for heart failure was discontinued following ablation if cardiac chambers returned to normal, the risk of right ventricular failure was reduced (e.g., reduced dimensions and improved distensibility of the caudal vena cava), and no abdominal fluid accumulation was observed. Treatment was adjusted based on follow-up cardiological examinations, and it was possible to discontinue medication between 3 and 12 months after the ablation procedure.

Prior to the electrophysiological study, all dogs (14/14; 100%) had received antiarrhythmic therapy, including sotalol in 7/14 cases (50%), diltiazem in 2/14 (14%), propafenone in 2/14 (14%), and amiodarone in 5/14 (36%). Combination therapy consisted of propafenone with sotalol in 1/14 cases (7%), sotalol with amiodarone in 1/14 dogs (7%), and diltiazem with amiodarone in 1/14 cases (7%). In all dogs, pharmacological treatment remained ineffective despite modification of the therapeutic regimen.

Among the dogs included in the study, the indications for ablation were OAVRT in 7/14 dogs (50%), ventricular ectopy (VE) associated with ventricular tachycardia (VT) in 4/14 dogs (28.6%), and focal atrial tachycardia (FAT) in 3/14 dogs (21.4%). One of seven dogs (14%) with OAVRT demonstrated electrocardiographic evidence of pre-excitation, manifested as a shortened PR interval (55 ms) without a visible delta wave. Recommended Holter monitoring follow-up was performed in 10 of 14 dogs at 1 month after ablation, in 8 of 14 dogs at 6 months after ablation, and in 7 of 14 dogs at 12 months after ablation (Table 2).

**Figure 1:**
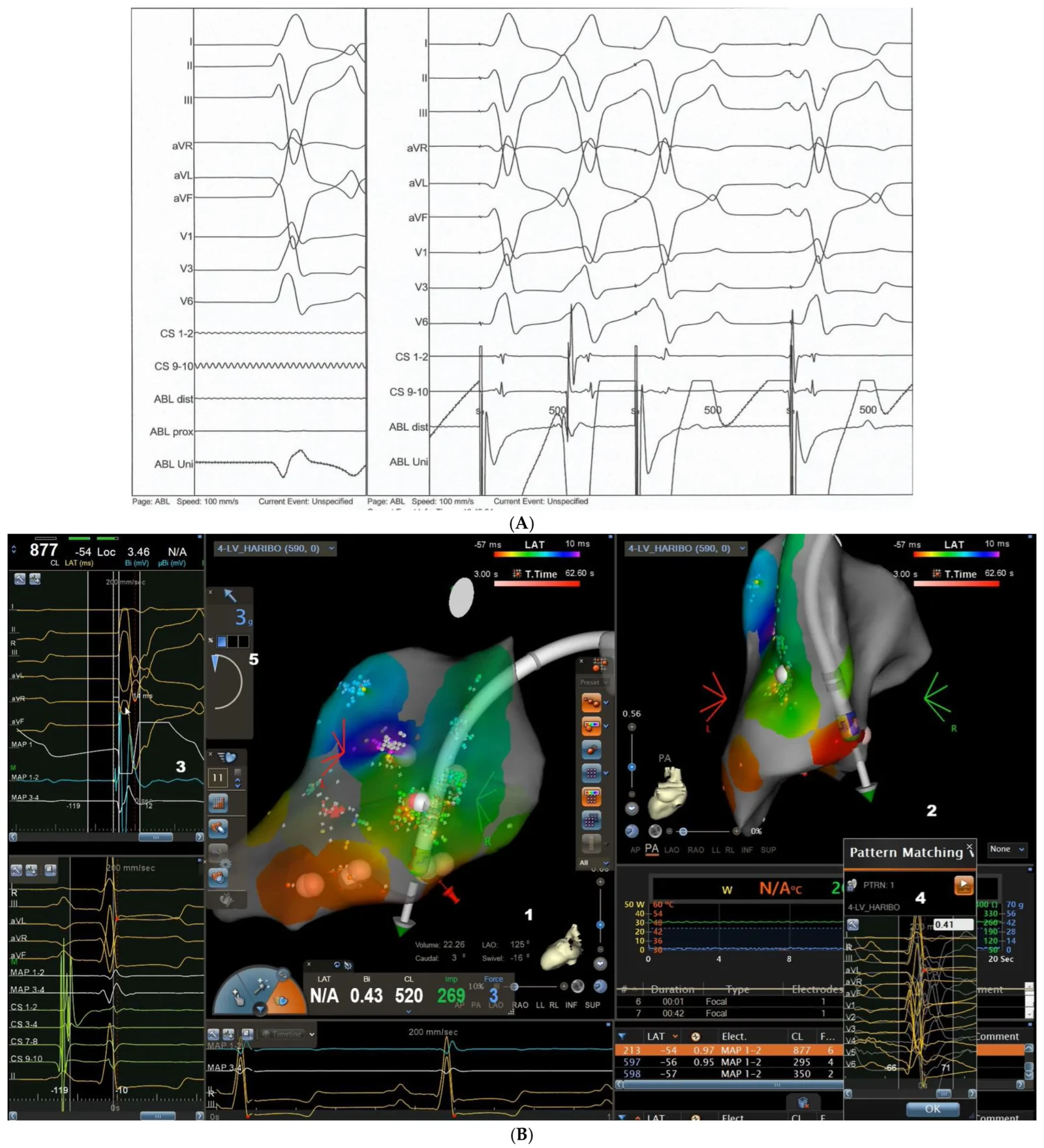
Ablation of a dog presenting left ventricular incessant salvos of nsVT. (**A**) QRS pattern of “clinical” VT (left panel) and paced ventricular beats (1st and 4th evolution) suggesting left basal posteroseptal origin (right panel). Paper speed: 100 mm/s; calibration: 1 mV/10 mm. (**B**) The position of the ablation catheter in the 3D CARTO LV map corresponds to the place where QRS morphology was optimal during pace mapping, and ablation of the VT substrate was successful. 3D CARTO (Panel 1 and 2) map, in the left lateral (LL) view of the left ventricle (1) and the posterior view of the left ventricle (2). Two orthogonal projections of the map support operation with precise and near-zero fluoro navigation inside the heart. Colors on the map represent Local Activation Time (LAT) measured as the earliest activation signal on the mapping catheter vs. nsVT. The red pin corresponds with the earliest activation, which is located on the base of the posteromedial papillary muscle. Signals from the ablation catheter—pointed to the earliest spot—are described on panel 3; it is the measured signal preceding on Map 1-2 vs. the beginning of nsVT QRS (−34ms). Channel MAP 1 represents a unipolar signal from the earliest spot showing a steep negative deflection, which is earlier than nsVT QRS and corresponds with the bipolar MAP 1-2 signal. Mapping and ablation were performed with the ThermoCool SmartTouch—contact force sensing catheter. Value (3 g) and vector of contact force on the map are measured from the spot where RF energy was delivered (Panel 5). Panel 4—pattern matching window—with a saved nsVT pattern, used to create an activation map based on at least 95% matching. Pattern matching was used for pace mapping to confirm the exact exit of arrhythmia before RF energy was delivered. Ablation RF was delivered early in the spot marked by the red pin, terminating nsVT.

In dogs with OAVRT, all accessory pathways (APs) were right-sided (7/7, 100%): 5/7 (71.4%) were located in the right posterior region and 2/7 (28.6%) in the right posteroseptal region. The origin of the VE was the outflow tract of the left ventricle (2/4), the apex of the left ventricle (1/4), and the posterior papillary muscle (1/4) (Figure 1). In three cases, left ventricular pace-mapping was performed by introducing an electrode through the femoral artery and the aortic valve. In one dog, access to the left ventricle was obtained via a transseptal puncture of the interatrial septum followed by advancement across the mitral valve. The site of origin of FAT was the crista terminalis in 2/3 dogs (66.7%). One dog was found to have numerous low-potential zones in the area of the interatrial septum, the ostium of the coronary sinus, and the roof of the right atrium. RFCA was ineffective in this dog despite the use of several RF application lines. The owners did not consent to re-ablation. RFCA was successful in 13 out of 14 cases, but in 3 cases, re-ablation was required. A re-ablation was performed 3 months after the initial procedure in one dog with a right posteroseptal AP, as a transient third-degree atrioventricular block occurred during the first procedure. Re-ablation was also performed 1 month after the initial procedure in one dog with FAT originating from the crista terminalis and 3 months after the initial procedure in one dog with VE originating from the left ventricular outflow tract.

Detailed data on the time points and the results of the cardiological examinations are set out in Table 2.

**Figure 2:**
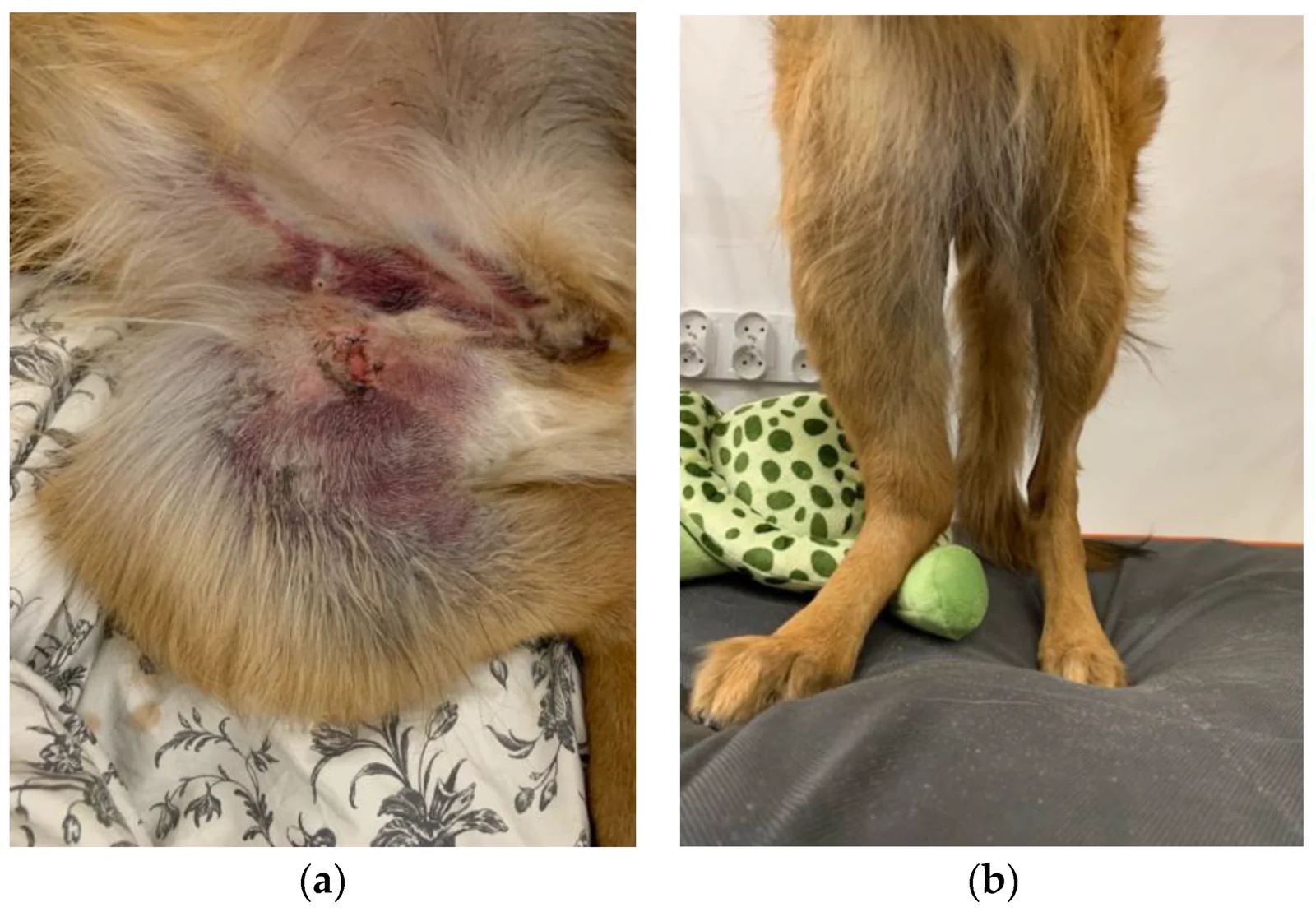
Hematoma after arterial puncture. (**a**) Visible wound site after removal of the vascular sheath and hematoma resulting from mechanical damage to the suture by the dog. (**b**) Swelling of the affected limb.

Complications occurred in two dogs: transient third-degree atrioventricular block and a hematoma in the lower extremity following an intra-arterial puncture in a German Shepherd (Figure 2). The atrioventricular block resolved spontaneously within 3 min of onset, did not require temporary or permanent pacing, and was attributable to ablation of the accessory pathway in close proximity to the atrioventricular node. The hematoma was managed conservatively and resolved after approximately 4 weeks.

## 4. Discussion

We describe a single-center experience with RFCA in dogs diagnosed with tachyarrhythmias of three distinct mechanisms: AP-mediated OAVRT, FAT, and ventricular arrhythmias, the last defined here as ventricular ectopy associated with ventricular tachycardia. Across this heterogeneous group, RFCA was feasible, and neither major nor permanent complications arose. Yet the three subtypes resist joint discussion. Mechanism, mapping strategy, procedural complexity, expected success, and prognosis differ, and each demands its own reading of the results.

Outcomes were most consistent in the OAVRT subgroup, which comprised 7 of 14 dogs. All APs were right-sided—most in the right posterior region, the remainder right posteroseptal. This distribution matched that reported in larger canine series [17,18] while differing from the patterns typically reported in humans [28,29]. Clinically, this subgroup matters because AP-mediated OAVRT may be incessant and lead to TICM, which was present before ablation in three of these seven dogs. Follow-up ranged from 1 to 78 months, and only one dog required a second procedure; in that animal, the initial attempt was abandoned after transient third-degree atrioventricular block developed during ablation of a right posteroseptal AP. Posteroseptal targets lie close to the normal conduction system, and this case is a reminder that energy delivery and mapping precision cannot be compromised in that region. Taken together, AVRT emerged as the most predictable indication in our series, provided the AP could be clearly localized and safely targeted.

Responses in the FAT subgroup (3 of 14 dogs) were considerably less uniform. Two dogs had an arrhythmogenic focus in the crista terminalis; the third had a far more complex atrial substrate, with multiple low-potential zones involving the interatrial septum, the ostium of the coronary sinus, and the right atrial roof. This distinction has important clinical implications. A discrete focus lends itself to focal ablation, whereas extensive atrial abnormality demands broader mapping, multiple RF applications, or repeat procedures and offers a lower likelihood of complete arrhythmia elimination. One dog with FAT required re-ablation, and RFCA failed in the dog with widespread low-potential atrial areas, whose owners declined a second attempt. FAT should therefore be interpreted as a heterogeneous arrhythmia rather than a single, uniform indication for RFCA. When the arrhythmia arises from a discrete ectopic focus, targeted ablation may be expected to have a more favorable outcome. In contrast, cases associated with a more diffusely abnormal atrial substrate (which may be considered multifocal atrial tachycardia) may be less predictable. This is in line with previous reports of right-sided FAT in dogs and with observations of multiple arrhythmogenic foci in some patients [9,13].

In contrast to supraventricular tachyarrhythmias, ventricular arrhythmias remain a more challenging and less extensively explored target for interventional treatment. This may be related to the greater diversity of mechanisms underlying these arrhythmias, their frequent association with structural or functional myocardial abnormalities, and the potential risks associated with ventricular mapping and ablation [11,30]. In our experience, the VA subgroup posed the greatest procedural challenge. Although it included only four of the 14 dogs, the ectopic foci were located in technically demanding regions, including the left ventricular outflow tract, left ventricular apex, and posterior papillary muscle. Three of the four had TICM, confirming that these arrhythmias were clinically significant rather than incidental electrocardiographic findings. Unlike OAVRT and FAT, VA ablation requires extensive ventricular mapping and, for left-sided foci, arterial retrograde aortic or transseptal access. These requirements have important implications for both procedural feasibility and safety. One dog required re-ablation, and the single vascular complication in this study, a hematoma following intra-arterial puncture, developed after an otherwise successful ablation.

The results obtained by our team are consistent with those reported in the literature [11,20,21,25]. In four dogs in which we performed ablation of ventricular arrhythmias, the ectopic foci could be successfully targeted. This resulted in complete resolution of clinically relevant arrhythmia in three dogs, in which arrhythmia burden decreased after RFCA from >60% of the recording to clinically insignificant levels, accounting for <5%, and a marked reduction in the burden and complexity of ventricular arrhythmias in one dog, in which Holter monitoring performed one month after RFCA showed a 25% reduction in arrhythmia burden, with no recorded episodes of non-sustained VT.

These findings must nevertheless be interpreted with caution because of the small number of dogs with ventricular arrhythmias eligible for RFCA treatment. With only four dogs treated, the study cannot provide a reliable estimate of procedural success or support firm conclusions regarding efficacy. Interpretation is further limited by the absence of a control group, differences in the underlying arrhythmogenic substrate, and variation in follow-up duration and methodology. The partial response observed in one dog indicates that ablation of the predominant ectopic focus may not always result in complete arrhythmia suppression. Possible explanations include additional arrhythmogenic foci, an intramural or otherwise inaccessible site of origin, incomplete lesion formation, or diffuse underlying myocardial disease. Thus, these findings primarily demonstrate that ventricular arrhythmia mapping and ablation are technically feasible in dogs and may have clinical value in selected patients, particularly when arrhythmias are symptomatic, clinically significant, inadequately controlled with antiarrhythmic drugs, or associated with unacceptable treatment-related adverse effects. Larger prospective studies are required to determine procedural and long-term success rates, assess recurrence and complications, identify predictors of response, and define the role of catheter ablation in canine ventricular arrhythmia management.

When the study population is considered as a whole, outcomes differed according to arrhythmia mechanism. A well-defined accessory pathway mediating OAVRT represented the most predictable indication, with a high procedural success rate and little need for reintervention. Outcomes in FAT depended on whether the arrhythmia originated from a discrete focal source. Ventricular arrhythmia ablation, although promising in selected dogs, was the most technically complex procedure in our experience, particularly for left-sided foci and those located near critical anatomical structures, including the conduction system, papillary muscles, and ventricular outflow tracts. Accordingly, the overall success rate of 13 of 14 procedures should be interpreted cautiously, as it combines outcomes from distinct arrhythmia mechanisms and should not be extrapolated to any individual arrhythmia subtype.

Every dog in this study had received antiarrhythmic treatment before RFCA, and pharmacological therapy was judged ineffective despite adjustment of the regimen, which speaks to the clinical role of RFCA in dogs with persistent or recurrent tachyarrhythmias. Rhythm control matters, especially where TICM is present, since sustained tachyarrhythmia drives myocardial dysfunction that is potentially reversible. TICM affected 7 of 14 dogs here and was distributed across all three subtypes. Although OAVRT is a well-recognized cause, TICM is evidently not confined to AP-mediated supraventricular tachycardia. Its occurrence in dogs with ventricular arrhythmias argues for weighing arrhythmia burden and chronicity when selecting candidates for ablation.

Three-dimensional electroanatomical mapping (3D EAM) forms another strand of this experience. The CARTO system was used in the three most recent dogs: two AP ablations and one VA ablation, with diagnostic and ablation catheters positioned without fluoroscopy. Three cases permit no formal comparison with conventional procedures, but the impression is that 3D EAM aids anatomical orientation, facilitates substrate localization, and reduces reliance on fluoroscopy. The benefit may be greatest in VA ablation, where precise localization of the ectopic focus is essential, and fluoroscopic landmarks alone may not suffice. The feasibility of 3D EAM in canine electrophysiology and ablation has been described previously [20,21,22,23,25,26]. To the best of our knowledge, this was the first successful ablation of a ventricular arrhythmia focus using the CARTO 3D EAM.

In our series, the safety profile of the procedures was acceptable. Complications were observed in two dogs and included transient third-degree atrioventricular block during AP ablation and a post-procedural hematoma after intra-arterial puncture in a dog with otherwise successful ablation. Neither complication was permanent, but both are clinically relevant. Transient atrioventricular block reflects the proximity of posteroseptal accessory pathways to the compact atrioventricular node and the associated risk of injury to the normal conduction system during energy delivery. The hematoma is attributable to arterial vascular access, which is required more frequently in left-sided ventricular procedures. Both argue for careful case selection, deliberate planning of vascular access, peri-operative monitoring, and operators experienced in electrophysiological mapping as well as in managing procedural complications.

An important limitation of RFCA for ventricular arrhythmias in dogs may be the need to ablate ectopic foci located within the left ventricle. In small breed dogs, vascular access may be considerably more difficult, or even technically impossible, due to the small size of the peripheral arteries and the diameter of the vascular sheaths required for the procedure. In such cases, transseptal puncture with access to the left ventricle through the left atrium and mitral valve may be necessary. However, this approach carries a higher risk of potential complications compared with the standard arterial approach and also requires additional, costly equipment. Further studies, including larger patient groups and longer follow-up, are needed to better define the efficacy, safety, and clinical relevance of RFCA in this group of arrhythmias [20,21,22,23,25,26].

## 5. Limitations

Several limitations should be considered when interpreting the results of our study. First, the number of dogs was relatively small, which limits the strength and generalizability of the conclusions. This is particularly relevant for patients with ventricular arrhythmia, as RFCA was performed in only four dogs with such a diagnosis. Although the results were promising, this small number of dogs does not allow for reliable estimates of procedural success, recurrence, or long-term efficacy. The study population was also heterogeneous. The dogs differed with respect to the type of arrhythmia, underlying cardiac disease, clinical presentation, and prior antiarrhythmic treatment. While this reflects the diversity of cases encountered in clinical practice, it also hampers direct comparisons between patients. The absence of a control group constitutes a further limitation, as it precludes a meaningful comparison with alternative treatment strategies, including pharmacological therapy alone.

Another limitation is the retrospective nature of the study. Because the analysis was based on medical records collected over time, the diagnostic work-up, data completeness, and follow-up were not fully standardized across all cases. The available information depended on the diagnostic tests performed in individual dogs, including echocardiographic assessment, cardiac biomarker measurements, and assessment of electrocardiographic records. In addition, the diagnostic criteria and arrhythmia classification were based on the available 6- and 12-lead ECG recordings, which may have varied between patients and across the study period.

Moreover, the dogs were referred to our center from various regions of Poland, which precluded standardized follow-up performed by members of our team. Because echocardiographic, electrocardiographic, and Holter re-evaluations were in some cases carried out at the referring practices rather than at our institution, these follow-up examinations were conducted by different operators using different equipment and were not performed according to a uniform protocol. Moreover, in some dogs we received only reported results of the follow-up examinations, without access to the underlying recordings or raw data, which for the reasons outlined above precluded any independent analysis on our part. As a result, we were unable to directly compare the follow-up cardiac findings with the baseline examinations obtained at our center, which limits the assessment of post-procedural changes in echocardiographic parameters, arrhythmia burden on Holter monitoring, and other indicators of procedural efficacy over time.

In summary, these factors mean that the results should be interpreted with appropriate caution. The cases of arrhythmia treated with RFCA presented here demonstrate that RFCA may provide clinical benefit in certain types of cardiac rhythm disturbances. This applies in particular to ventricular arrhythmias, for which the current state of knowledge remains limited. Future prospective studies involving larger numbers of dogs, standardized follow-up protocols, and collaboration between referring centers will be essential to better define procedural efficacy, recurrence rates, complications, and the long-term role of RFCA in the management of arrhythmias in dogs.

We believe that there is still a problem with the diagnosis of patients requiring electrophysiological evaluation and catheter ablation. Many patients are incorrectly diagnosed with conditions such as primary dilated cardiomyopathy without further differential diagnostic investigation for TICM. The findings of our study should be generalized to the canine population with regard to the feasibility of catheter ablation for supraventricular and ventricular arrhythmias and the importance of differential diagnosis of the dilated cardiomyopathy phenotype.

## 6. Conclusions

RFCA is an effective treatment for tachyarrhythmias in dogs, especially in supraventricular arrhythmias involving accessory pathways. The available literature indicates that RFCA can be highly effective in AVRT, with resolution of clinical signs and improvement of cardiac function in dogs affected by TICM. For FAT and atrial flutter, the evidence is more limited, although the reported outcomes suggest that ablation may be a useful treatment option in selected cases. RFCA may be a valuable therapeutic option in selected dogs with symptomatic, drug-refractory ventricular arrhythmias; however, this method cannot be considered a routine treatment approach for this type of arrhythmia. Nevertheless, RFCA should still be regarded as a specialist procedure, requiring careful case selection, electrophysiological experience, and appropriate anesthetic and technical facilities. Electroanatomical systems may improve the safety and effectiveness of ablation and could represent an important direction for the future development of electrophysiological studies and arrhythmia ablation procedures in dogs.

## Figures and Tables

**Table 1:** Population characteristics. AVRT—atrioventricular re-entry tachycardia; FAT—focal atrial tachycardia; VE—ventricular ectopy.

Breed	Sex [F/M]	Age [years]	Body Weight [kg]	Arrhythmia	TICM [Y/N]	Follow Up [months]	Redo [Y/N]
French Bulldog	M	8	10	FAT	N	36	Y
Labrador	M	3	27	FAT	Y	0	N
Doberman	M	6	43	FAT	N	24	N
Labrador	M	9	33	AVRT	Y	38	Y
Golden Retriever	M	1	28	AVRT	N	54	N
Australian Shepherd	F	2	22	AVRT	N	48	N
Labrador	F	3	42	AVRT	N	29	N
Boxer	F	1	22	AVRT	Y	12	N
English Bulldog	M	3	29	AVRT	N	1	N
Yakutian Laika	M	2	23	VE	Y	78	Y
Hungarian Pointer	M	1	22	VE	N	56	N
German Shepherd	F	2	30	VE	Y	75	N
Mixed breed	M	0.75	23	VE	Y	1	N

**Table 2:** Characteristics of the dogs before and after the ablation procedure at each time point, including echocardiographic, electrocardiographic, and Holter monitoring evaluations, as well as treatment before and after the procedure. SR—sinus rhythm, SVT—supraventricular tachycardia.

Breed	Diagnosed Arrhythmia	Echo Pre-Procedure	Echo 1 Month After Procedure	Echo 6 Months After Procedure	Echo 12 Months After Procedure	ECG Pre-Procedure	ECG 7 Days After Procedure	ECG 1 Month After Procedure	ECG 6 Months After Procedure	ECG 12 Months After Procedure	Holter Pre-Procedure	Holter 1 Month After Procedure	Holter 6 Months After Procedure	Holter 12 Months After Procedure	Treatment Directly Before Procedure	Treatment 1 Month After Procedure	Treatment 6 Months After Procedure	Treatment 12 Months After Procedure
French Bulldog	FAT	unremarkable	unremarkable	unremarkable	unremarkable	SVT	N/A	SR	SR	SR	N/A	SR	SR	SR	amiodarone	without any treatment	without any treatment	without any treatment
Labrador	OAVRT	TICM	N/A	unremarkable	unremarkable	SVT	SR	SR	SR	SR	SR with paroxysmal SVT, paroxysmal VPE	SR	SR	SR	pimobendan, torasemide, spironolactone, benazepril, diltiazem, amiodarone	pimobendan, benazepril, spironolactone	without any treatment	N/A
Labrador	OAVRT	TICM	TICM	unremarkable	unremarkable	SVT	SR	SVT	SR	SR	N/A	N/A	SR	N/A	pimobendan, sotalol	pimobendan, sotalol	without any treatment	without any treatment
Yakutian Laika	VE	TICM	TICM	unremarkable	unremarkable	VE	N/A	VE	SR	SR	VE	VE	SR	N/A	sotalol	sotalol	without any treatment	without any treatment
Hungarian Pointer	VE	unremarkable	unremarkable	unremarkable	unremarkable	VE	N/A	SR	SR	SR	VE	SR	SR	SR	propafenone	without any treatment	without any treatment	without any treatment
Labrador	FAT	TICM	N/A	N/A	N/A	SVT	SR	N/A	N/A	N/A	SVT	N/A	N/A	N/A	amiodarone, pimobendan, spironolactone, torasemide, benazepril	N/A	N/A	N/A
Golden Retriever	OAVRT	unremarkable	unremarkable	unremarkable	unremarkable	SVT	SR	SR	SR	SR	SVT	SR	SR	SR	without any treatment	without any treatment	without any treatment	without any treatment
German Shepherd	VE	TICM	unremarkable	unremarkable	unremarkable	VE	N/A	SR	SR	SR	N/A	SR	N/A	N/A	sotalol	without any treatment	without any treatment	without any treatment
Australian Shepherd	OAVRT	TICM	TICM	unremarkable	unremarkable	SR, paroxysmal SVT	SR	SR	SR	SR	SR, paroxysmal SVT arrhythmia burden: 80% of monitoring time	N/A	N/A	SR	pimobendan, furosemide, spironolactone, diltiazem	pimobendan	without any treatment	without any treatment
Doberman	FAT	unremarkable	unremarkable	unremarkable	unremarkable	SR, paroxysmal SVT susp. FAT	SR, SVEB	SR, paroxysmal SVT susp. FAT	SR, paroxysmal SVT susp. FAT	SR, paroxysmal SVT susp. FAT	SR, paroxysmal SVT arrhythmia burden: 40% of monitoring time	SR, paroxysmal SVT arrhythmia burden: 25% of monitoring time	SR, paroxysmal SVT arrhythmia burden: 15% of monitoring time	SR, paroxysmal SVT arrhythmia burden: 20% of monitoring time	sotalol + propafenone	sotalol + propafenone	sotalol + propafenone	sotalol + propafenone
Labrador	OAVRT	unremarkable	unremarkable	unremarkable	unremarkable	SVT	SR	SR	SR	SR	SVT	N/A	N/A	N/A	sotalol	without any treatment	without any treatment	without any treatment
Boxer	OAVRT	TICM	TICM	unremarkable	unremarkable	SR, paroxysmal SVT susp. AVRT	SR	SR	SR	SR	SR, paroxysmal SVT; arrhythmia burden: 90% of monitoring time	SR	SR	SR	pimobendan, sotalol	pimobendan	without any treatment	without any treatment
English Bulldog	OAVRT	unremarkable	unremarkable	N/A	N/A	sustained SVT	SR	SR	N/A	N/A	SR, paroxysmal SVT; arrhythmia burden: 50% of monitoring time	SR	N/A	N/A	sotalol + amiodarone	without any treatment	N/A	N/A
Mixed breed	VE	TICM	TICM	N/A	N/A	monomorphic ventricular arrhythmias: VT, ventricular couplets, ventricular triplets, bigeminy, VPC	SR, couplets, triplets	Monomorphic ventricular arrhythmias: ventricular couplets, ventricular triplets, bigeminy, VPC	N/A	N/A	SR, paroxysmal monomorphic ventricular arrhythmias: VT, ventricular couplets, ventricular triplets, bigeminy, VPC, arrhythmia burden: 90% of monitoring time	SR, monomorphic ventricular arrhythmias: ventricular couplets, ventricular triplets, bigeminy, VPC, arrhythmia burden: 65% of monitoring time	N/A	N/A	pimobendan + amiodarone	pimobendan + amiodarone + propafenone	N/A	N/A

## Data Availability

The original contributions presented in this study are included in the article. Further inquiries can be directed to the corresponding authors.

## Abbreviations

The following abbreviations are used in this manuscript:

RFCA	Radiofrequency Catheter Ablation
EPS	Electrophysiological Study
3D EAM	Three-dimensional Electroanatomical Mapping
TICM	Tachycardia-induced Cardiomyopathy
AVRT	Atrioventricular Reciprocating Tachycardia
VE	Ventricular Extrasystoles
VT	Ventricular Tachycardia
FAT	Focal Atrial Tachycardia
AP	Accessory Pathway
ARVC	Arrhythmogenic Right Ventricular Cardiomyopathy
RVOT	Right Ventricular Outflow Tract
VPCs	Ventricular Premature Contractions
